# Obestatin Accelerates the Healing of Acetic Acid-Induced Colitis in Rats

**DOI:** 10.1155/2016/2834386

**Published:** 2015-12-20

**Authors:** Aleksandra Matuszyk, Piotr Ceranowicz, Zygmunt Warzecha, Jakub Cieszkowski, Joanna Bonior, Jolanta Jaworek, Beata Kuśnierz-Cabala, Peter Konturek, Tadeusz Ambroży, Artur Dembiński

**Affiliations:** ^1^Department of Physiology, Jagiellonian University Medical College, 16 Grzegorzecka Street, 31-531 Cracow, Poland; ^2^Department of Anatomy, Jagiellonian University Medical College, 12 Kopernika Street, 31-034 Cracow, Poland; ^3^Department of Medical Physiology, Faculty of Health Sciences, Jagiellonian University Medical College, 12 Michałowskiego Street, 31-126 Cracow, Poland; ^4^Chair of Clinical Biochemistry, Department of Diagnostics, Jagiellonian University Medical College, 15a Kopernika Street, 31-501 Cracow, Poland; ^5^Department of Internal Medicine II, Thuringia Clinic, Teaching Hospital of the University of Jena, Rainweg 68, 07318 Saalfeld, Germany; ^6^Faculty of Physical Education and Sport, University of Physical Education, 78 Jan Paweł II Street, 31-571 Cracow, Poland

## Abstract

Obestatin, a 23-amino acid peptide derived from the proghrelin, has been shown to exhibit some protective and therapeutic effects in the gut. The aim of present study was to determine the effect of obestatin administration on the course of acetic acid-induced colitis in rats. *Materials and Methods*. Studies have been performed on male Wistar rats. Colitis was induced by a rectal enema with 3.5% acetic acid solution. Obestatin was administered intraperitoneally twice a day at a dose of 8 nmol/kg, starting 24 h after the induction of colitis. Seven or 14 days after the induction of colitis, the healing rate of the colon was evaluated. *Results*. Treatment with obestatin after induction of colitis accelerated the healing of colonic wall damage and this effect was associated with a decrease in the colitis-evoked increase in mucosal activity of myeloperoxidase and content of interleukin-1*β*. Moreover, obestatin administration significantly reversed the colitis-evoked decrease in mucosal blood flow and DNA synthesis. *Conclusion*. Administration of exogenous obestatin exhibits therapeutic effects in the course of acetic acid-induced colitis and this effect is related, at least in part, to the obestatin-evoked anti-inflammatory effect, an improvement of local blood flow, and an increase in cell proliferation in colonic mucosa.

## 1. Introduction

Obestatin is a 23-amino acid peptide derived from preproghrelin, a common prohormone for ghrelin and obestatin [1–3]. Obestatin was originally extracted from the rat stomach and the stomach seems to be a major source of circulating obestatin [1, 2, 4]. Secretion of obestatin is pulsative and displays an ultradian rhythmicity similar to ghrelin and growth hormone [5]. In contrast to ghrelin, obestatin has been reported to be an anorexic hormone, reducing food intake, gastric emptying time, jejunal motility, and body weight gain [2, 3].

Previous studies have shown that pretreatment with ghrelin, an alternative product of posttranslational processing of preproghrelin, protects gastric mucosa against damage evoked by different noxious factors [6–8] and inhibits the development of experimental acute pancreatitis [9, 10]. Moreover, apart from its protective effect, ghrelin exhibits therapeutic effect in the gut. It accelerates the healing of gastric [11], duodenal [11, 12] and oral ulcers [13], and colonic inflammation [14, 15]. Therapeutic effect of ghrelin has been also shown in experimental models of acute pancreatitis [16–19].

In the case of obestatin, there are studies which have shown that also this peptide exhibits some protective and therapeutic effects in the gut [3]. It has been demonstrated so far that preventive administration of obestatin inhibits the development of cerulein- and ischemia/reperfusion-induced acute pancreatitis [20, 21]. Moreover, Granata et al. have reported that obestatin promotes survival of pancreatic islets, especially *β*-cell [22]. In the stomach, it has been shown that treatment with obestatin accelerates the healing of acetic acid-induced gastric ulcers [23]. Moreover, previous studies have suggested that endogenous and exogenous obestatin may affect or be related to the development of colitis. Alexandridis et al. have found that the ratio of serum level of obestatin to ghrelin in patients with active inflammatory bowel disease (IBD) is significantly lower than in patients with remission [24]. This observation was confirmed by Jung et al. and they have suggested that the obestatin/ghrelin ratio may be useful in monitoring of remission in the course of IBD [25]. Furthermore, one experimental study has shown protective effect of obestatin in the colon. Pamukcu et al. have reported that administration of obestatin before and during the development of dextran sodium sulfate-induced colitis reduces the severity of this inflammation [15].

The objective of the present research was to determine whether administration of obestatin after the development of colitis exhibits therapeutic effect in this disease.

## 2. Material and Methods

### 2.1. Animals and Treatment

Studies were performed on 80 male Wistar rats weighing 270–320 g and were conducted following the experimental protocol approved by the 1st Local Committee of Ethics for the Care and Use of Laboratory Animals in Cracow (Permit number 2/2013 released on January 16, 2013). During the experiments animals were kept in cages placed in room temperature with a 12 h light-darkness cycle. Animals were fasted, with free access to water, for 18 h prior to induction of colitis. Later, water and food were available* ad libitum*.

The animals were randomly divided into four groups: (1) control rats without colitis induction treated intraperitoneally (i.p.) with saline; (2) rats without colitis induction treated i.p. with obestatin; (3) rats with colitis treated i.p. with saline; (4) rats with colitis treated i.p. with obestatin.

In the rats anesthetized with pentobarbital (30 mg/kg i.p., Vetbutal, Biowet, Puławy Poland), colitis was induced by a rectal enema with 1 mL of 3.5% (v/v) acetic acid diluted in saline. Acetic acid solution was administered through a polyethylene catheter inserted into the rectum. There are different models of acetic acid-induced colitis and the tip of catheter can be positioned from 1.2 [26] to 8 cm [27] proximal to the anus verge. For this reason we have chosen an intermediate depth of catheter insertion, 4.5 cm from the anus. Rats without induction of colitis obtained rectal enema with an aqueous saline solution administered at the same manner as a solution of acetic acid in animals with induction of colitis.

Starting 24 hours after a rectal enema with saline or acetic acid, the rats were treated with saline (groups 1 and 3) or obestatin (groups 2 and 4) administered i.p. twice a day. Rat obestatin (Yanaihara Institute, Shizuoka, Japan) was given at a dose of 8 nmol/kg. This dose was chosen because previous studies have shown that obestatin given at a dose 8 nmol/kg exhibits strong and repeatable therapeutic effect in the healing of chronic gastric ulcers [23]. Rats from each experimental group were randomly divided into two subgroups. In the first subgroup, the healing rate of the colon was evaluated 7 days after the acetic acid enema. In the second subgroup evaluation was performed 7 days later. Each subgroup consisted of 10 animals.

### 2.2. Measurement of Colonic Blood Flow and Colonic Damage

At the end of the experiments, animals were anesthetized again with pentobarbital. After opening the abdominal cavity and exposure of the colon, the rate of colonic blood flow was measured using laser Doppler flowmeter (PeriFlux 4001 Master monitor, Perimed AB, Järfälla, Sweden), in accordance with the methodology described before [28]. The measurement of mucosal blood flow was performed every time in five parts of the descending and sigmoid colon and the main value of five records was expressed as the percentage of the value obtained in the animals from the control group. After the measurement of colonic blood flow, anesthetized animals were euthanized by exsanguination from the abdominal aorta. Then, the area of mucosal damage was measured, using a computerized planimeter (Morphomat, Carl Zeiss, Berlin, Germany), in accordance with the method described earlier [12]. Briefly, the large bowel was removed from the body, opened along of its long axis on the side opposite to the mesentery, and washed with ice-cold saline. The colon samples were flattened and carefully sandwiched between two layers of transparent plastic folder. The area of damage was measured by tracing its perimeter.

### 2.3. Biochemical Analysis

The dynamics of DNA replication in the colon mucous membrane was marked with radioisotope method through measurement of tritium-labeled thymidine incorporation into DNA, according to the methodology described before [29]. A mucous membrane sample was fragmented with scissors, subsequently, the tissue was incubated in 37°C for 45 minutes in 2 mL of nutritive solution containing tritium-marked thymidine ([6-^3^H] thymidine, 20–30 Ci/mmol; Institute for Research, Production and Application of Radioisotopes, Prague, Czech Republic), with activity of 8 *μ*Ci/mL. Incorporation of [^3^H]-thymidine into DNA was determined by counting 0.5 mL DNA-containing supernatant in liquid scintillation system. The rate of DNA synthesis was expressed as disintegration per minute [^3^H]-thymidine per microgram DNA (dpm/*μ*g DNA).

Samples of the colonic mucosa, in which the concentration of interleukin-1*β* was measured, were homogenized in phosphate buffer at 4°C. Then the homogenate was centrifuged and the concentration of interleukin-1*β* in the supernatant was determined using the Rat IL-1*β* Platinum Elisa (Bender MedSystem GmbH, Vienna, Austria). The concentration of interleukin-1*β* of the colonic mucosa was expressed in nanograms per 1 gram of tissue.

Biopsy samples for measurement of mucosal myeloperoxidase activity were homogenized in ice-cold potassium phosphate and, until the marking was done, stored at the temperature of −60°C. Marking myeloperoxidase activity was performed with the use of a modification of the method described by Bradley et al. [30]. Results obtained in units per gram of tissue were finally expressed as a percentage of the value observed in the control group.

### 2.4. Statistical Analysis

Statistical analysis of the data was carried out by one-way analysis of variance (ANOVA) followed by Tukey's multiple comparison test using GraphPadPrism (GraphPad Software, San Diego, CA, USA). Differences were considered to be statistically significant when *P* was less than 0.05.

## 3. Results

**Figure 1:**
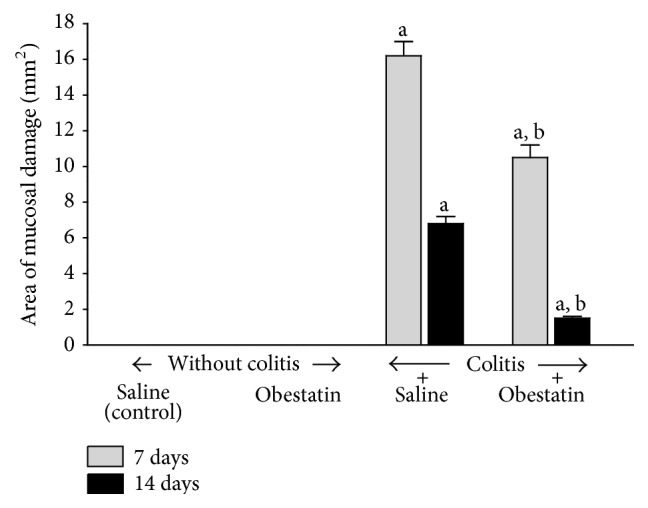
Effect of saline or obestatin given intraperitoneally for 7 or 14 days on the area of colonic lesions in rats without or with acetic acid-induced colitis. Mean value ± SEM. *N* = 10 animals in each experimental group and each time of observation. ^a^ *P* < 0.05 compared to control at the same time of observation; ^b^ *P* < 0.05 compared to colitis plus saline at the same time of observation.

Figure 1 shows the influence of obestatin administration on the healing of acetic acid-induced mucosal damage in the colon. In saline- and obestatin-treated rats without induction of colitis no mucosal damage was observed. In saline-treated rats, 7 days after the induction of colitis, the mucosal damage area was 16.2 ± 0.8 mm^2^, whereas 7 days later it was reduced to 6.8 ± 0.4 mm^2^ as a result of spontaneous regeneration. Treatment with obestatin for 7 days after administration of acetic acid accelerated a reduction in the mucosal damage area by 35.2%. After the next 7 days of treatment with obestatin, the area of colonic damage was reduced by around 78% when compared to lesions observed in the animals treated with saline. The healing promoting effect of obestatin was statistically significant at both periods of observation, after 7 and 14 days of obestatin administration (Figure 1).

**Figure 2:**
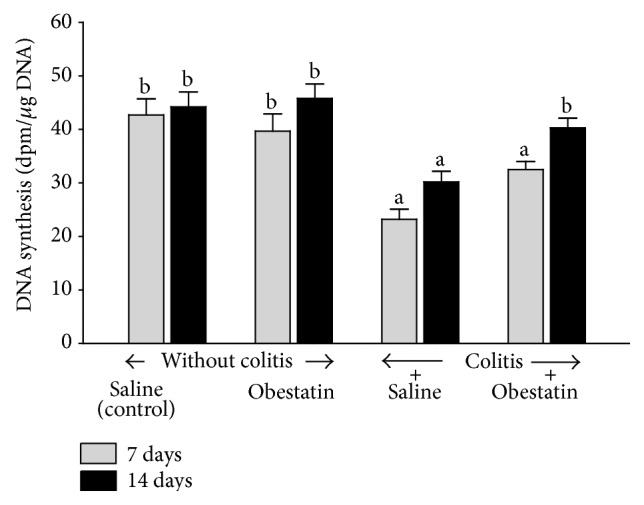
Effect of saline or obestatin given intraperitoneally for 7 or 14 days on the rate of DNA synthesis in colonic mucosa in rats without or with acetic acid-induced colitis. Mean value ± SEM. *N* = 10 animals in each experimental group and each time of observation. ^a^ *P* < 0.05 compared to control at the same time of observation; ^b^ *P* < 0.05 compared to colitis plus saline at the same time of observation.

In the rats without induction of colitis, administration of obestatin at a dose used failed to affect significantly DNA synthesis in colonic mucosa (Figure 2). Induction of colitis by an enema with acetic acid led to reduction in mucosal DNA synthesis in the colon. DNA synthesis in the colonic mucosa was significantly reduced by around 45 and 32% at the 7th and 14th day after induction of colitis, respectively. Treatment with obestatin partly reversed the colitis-evoked reduction in DNA synthesis in the colonic mucosa and this effect was statistically significant after 14 days of obestatin administration (Figure 2).

**Figure 3:**
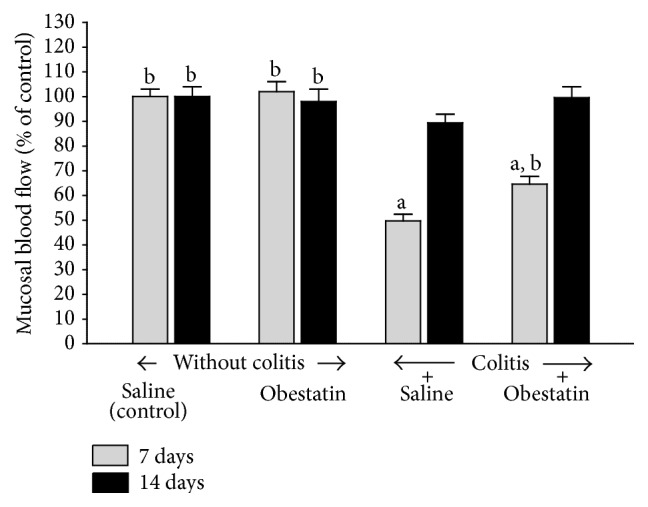
Effect of saline or obestatin given intraperitoneally for 7 or 14 days on mucosal blood flow in the colon rats without or with acetic acid-induced colitis. Mean value ± SEM. *N* = 10 animals in each experimental group and each time of observation. ^a^ *P* < 0.05 compared to control at the same time of observation; ^b^ *P* < 0.05 compared to colitis plus saline at the same time of observation.

In the groups of animals without induction of colitis, intraperitoneal administration of obestatin for 7 or 14 days failed to affect mucosal blood flow in the colon (Figure 3). In the rats with colitis, 7 days after an enema with acetic acid, blood flow through the colonic mucosa was significantly reduced by around 50%, when compared to the value observed in the control animals without colitis. After the next seven days, mucosal blood flow in the colon of the animals with colitis was almost fully restored and no significant difference was observed in comparison to a value in control group of animals. In the rats with colitis, administration of obestatin caused an improvement of mucosal blood flow in the colon and this effect was statistically significant at the 7th day after the induction of colitis (Figure 3).

**Figure 4:**
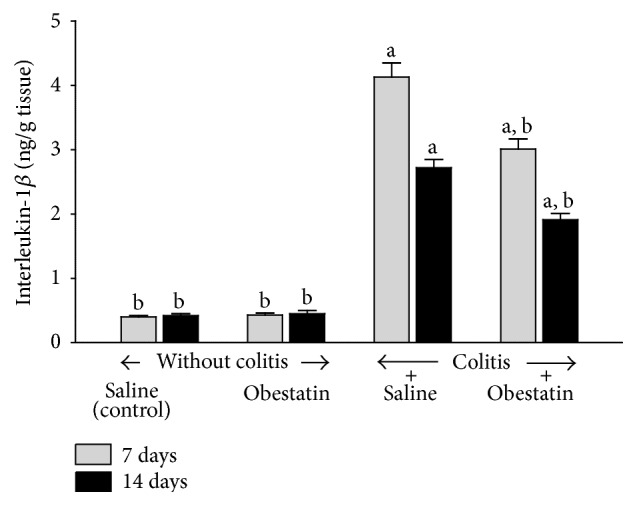
Effect of saline or obestatin given intraperitoneally for 7 or 14 days on interleukin-1*β* concentration in colonic mucosa in rats without or with acetic acid-induced colitis. Mean value ± SEM. *N* = 10 animals in each experimental group and each time of observation. ^a^ *P* < 0.05 compared to control at the same time of observation; ^b^ *P* < 0.05 compared to colitis plus saline at the same time of observation.

In the rats without colitis, administration of obestatin for 7 or 14 days at the dose used was without any effect on mucosal concentration of interleukin-1*β* (IL-1*β*) in the colon (Figure 4). Induction of colitis significantly increased mucosal concentration of IL-1*β* in the colon. As shown in Figure 4, rats with colitis demonstrated more than 10-fold and 6-fold increase in this parameter at the 7th day and 14th after induction of colitis, respectively. Administration of obestatin at the dose used partly reversed the colitis-evoked increase in mucosal concentration of IL-1*β* and this effect was statistically significant in both periods of observation (Figure 4).

**Figure 5:**
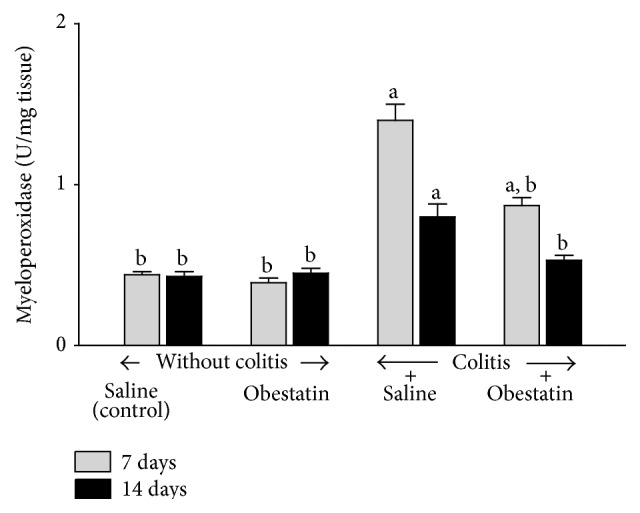
Effect of saline or obestatin given intraperitoneally for 7 or 14 days on myeloperoxidase activity in colonic mucosa in rats without or with acetic acid-induced colitis. Mean value ± SEM. *N* = 10 animals in each experimental group and each time of observation. ^a^ *P* < 0.05 compared to control at the same time of observation; ^b^ *P* < 0.05 compared to colitis plus saline at the same time of observation.

Administration of obestatin for 7 or 14 days was without effect on mucosal myeloperoxidase activity in the colon in the rats without colitis induction (Figure 5). In rats treated with saline after induction of colitis, 7 days after induction of this inflammation, myeloperoxidase activity in colonic mucosa was increased by 318%. Also, after the next 7 days, almost 2-fold increase in myeloperoxidase activity was still observed in colonic mucosa in rats with colitis treated with saline. Treatment with obestatin reduced the colitis-evoked increase in myeloperoxidase activity in the colonic mucosa. This effect was statistically significant in both periods of observation, 7 and 14 days after induction of (Figure 5).

## 4. Discussion

In this study, we have investigated the influence of treatment with obestatin on the course of acetic acid-induced colitis. We have found that intraperitoneal administration of obestatin given at the dose 8 nmol/kg significantly accelerates the recovery of colonic wall integrity in rats with this model of experimental colitis. Maintaining the integrity of the digestive tract mucous membrane depends on preservation of balance between proliferation and loss of epithelial cells. A disruption of that balance, as a result of a decrease in cell proliferation, apoptosis, and/or increased cell loss, leads to mucosal atrophy and/or ulceration [31, 32], whereas an increase in cell division may lead to mucosal hypertrophy. On the other hand, stimulation of cell proliferation typically leads to increase in the protection of the digestive tract mucous membrane against damaging factors and accelerates the healing of mucosal damage [33–35]. Synthesis of DNA is an indispensable process for mitosis to take place [36]. Therefore, assessment of DNA synthesis dynamics through measurement of incorporation of tritium-marked thymidine into cellular DNA reflects vitality of cells and cellular division dynamics.

In our present study, we have found that administration of obestatin in animals without induction of colitis failed to significantly affect mucosal DNA synthesis in the colon. This leads to the conclusion that, in animals with normal colonic mucous membrane, treatment with obestatin given at the dose of 8 nmol/kg does not stimulate DNA synthesis in colonic mucosa and therefore this peptide seems to be safe and does not bring a risk of hyperplasia and hypertrophy of colonic mucosa. On the other hand, administration of obestatin in rats with colitis led to a considerable improvement of mucosal DNA synthesis in the colon. This observation indicates that therapeutic effect of obestatin in the course of acetic acid-induced colitis is, at least in part, a result of increase in cell vitality in colonic mucosa.

Another factor that plays a role in maintaining mucosal integrity is suitable, adequate for the demand, organ blood flow. The important role of vascular mechanisms in protection and healing of gastrointestinal mucosa has been previously described [37–39]. Experimental studies have demonstrated that exposure of the stomach to damaging factors leads to small mucosal damage, as long as a sufficient blood flow is maintained. A similar significance of sufficient blood flow in maintaining mucosal integrity was also demonstrated in other parts of the digestive tract, including the oral cavity [13], esophagus [38], duodenum [11, 12] and colon [39]. In our present study, we have observed that intrarectal administration of acetic acid solution decreases mucosal blood flow in the colon. Seven days after induction of colitis mucosal blood flow was decreased by around 50% in comparison with a value observed in control group without colitis. After the next 7 days, blood flow in colonic mucosa in rats with colitis was still lower than in control group, but this difference was statistically insignificant.

Administration of obestatin in the dose used did not exhibit any influence on blood flow in the colonic mucous membrane in animals without colitis. In contrast to that, administration of obestatin in animals with colitis led to a significant improvement in blood flow in the colonic mucous membrane, and this effect was statistically significant at the 7th day after induction of colitis. After the next 7 days a value of blood flow in rats with colitis treated with obestatin was similar to that recorded in control animals without colitis. Moreover, this obestatin-evoked improvement of mucosal blood flow was connected with a decrease in the area and severity of colonic mucosal damage. This observation indicates that therapeutic effect of obestatin in acetic acid-induced colitis involves an improvement of mucosal blood flow in the large bowel.

The next interesting finding originated from our present study is the influence of intraperitoneal administration of obestatin on the concentration of interleukin-1*β* (IL-1*β*) and myeloperoxidase activity in colonic mucosa of animals with colitis. Activation of inflammatory cells with subsequent release of proinflammatory cytokines is responsible for intensity of local and systemic inflammatory response [40]. Between proinflammatory cytokines, IL-1*β* plays a fundamental role in the initiation of biochemical cascade of inflammation [40–45]. Results obtained in our present study have demonstrated that acetic acid-induced colitis led to a tenfold and sixfold increase in IL-1*β* concentration in colonic mucosa at the 7th an 14th day after induction of this inflammation, respectively. Intraperitoneal administration of obestatin partially but significantly reduced that increase. On the other hand, administration of obestatin failed to affect IL-1*β* concentration in the mucous membrane in rats without colitis.

Myeloperoxidase (MPO) is an enzyme which is present in azurophilic granules of neutrophil granulocytes. Antimicrobial function of neutrophils is related, among others, to their possibility to generate reactive oxygen species (ROS) during the respiratory burst [46]. On the other hand, excess of ROS leads to oxidative damage of own tissues on cellular and subcellular level resulting in destruction of proteins, nucleic acids, and lipids [46–49]. MPO is released by activated neutrophils and reflects the degree of tissue infiltration by those granulocytes [46, 50]. Those data are in agreement with our present observation. Seven days after rectal enema with acetic acid, we have observed a threefold increase in MPO activity in the colonic mucous membrane in rats treated with saline. After the next 7 days mucosal activity of MPO in colon of rats with colitis was still significantly increased above a level observed in control rats without induction of colitis. Intraperitoneal administration of obestatin in animals with acetic acid-induced colitis caused a considerable decrease in colonic MPO activity. In contrast to that, mucosal activity of MPO in the colon of rats without colitis was low and administration of obestatin was without effect on colonic MPO activity in those rats.

Our findings mentioned above concerning the inhibitory effect of treatment with obestatin on IL-1*β* concentration and MPO activity in colonic mucosa in rats with acetic acid-induced colitis indicate that the healing promoting effect in this kind of colitis is, at least in part, related to anti-inflammatory properties of obestatin. On the other hand, obestatin failed to affect mucosal IL-1*β* concentration and MPO activity in the colon of rats without induction of colitis. This observation suggests that obestatin does not disturb the immune system in normal circumstances, without inflammation.

As stated in the introduction, ghrelin and obestatin are peptides encoded by the same gene and derived from a common prohormone [1–3]. Ghrelin exhibits protective and therapeutic effect in various organs and experimental models of organ damage [6–19]. Data on the effects of obestatin are less numerous. Previous studies have demonstrated that this peptide demonstrates protective effect in the large bowel and its earlier application reduces damage in colitis induced by dextran sodium sulfate (DSS) [15]. Our present studies have shown that obestatin also exhibits a therapeutic effect in the course of colitis and administration of this peptide after the development of acetic acid-induced colitis accelerates the healing of inflammation. Differences between ghrelin and obestatin are based on their action mechanisms. Ghrelin acts directly via activation of GHS-R receptor and indirectly via a release of growth hormone and IGF-1 [2, 3, 51]. In the case of obestatin, its receptor is still unknown. Initially it was thought that obestatin acts by binding to the G protein-coupled receptor 39 (GRP39) [1], but later studies do not seem to confirm this hypothesis [52]. Also there is another effect of these peptides on food intake. Ghrelin stimulates food intake, whereas, in the case of obestatin, its inhibitory effect on appetite has been postulated [1–3].

There are numerous experimental models of inflammatory bowel disease, because each of these models is not perfect and does not fully meet the clinical condition. DSS-induced colitis is reproducible and one of widely used animal models of IBD. DSS mainly affects the large bowel, but some studies have reported that DSS also affects the distal parts of small intestine [27]. In mice or rats, administration of DSS causes hematochezia, body weight loss, shortening of intestine, mucosal ulcers, and infiltration of neutrophils [53]. DSS causes erosion with complete loss of surface epithelium because of its deleterious effect on epithelial cells [27]. Acetic acid-induced colitis is an animal model of colitis that shows close resemblance to human IBD in terms of pathogenesis, morphological features, and inflammatory factors involved in this kind of inflammation [27]. Intrarectal enema with acetic acid solution causes inflammation characterized by neutral infiltration into intestinal tissue, massive necrosis of mucosal and submucosal tissues, edema, vascular dilatation, and submucosal ulceration. Those findings are similar to that observed in human colitis [27].

In conclusion, we can say that treatment with obestatin accelerates the healing of acetic acid-induced colitis and this effect seems to be related to the obestatin-evoked anti-inflammatory effect, an improvement of local mucosal blood flow, and an increase in cell proliferation in colonic mucosa.
